# Kras activation in p53-deficient myoblasts results in high-grade sarcoma formation with impaired myogenic differentiation

**DOI:** 10.18632/oncotarget.3856

**Published:** 2015-05-15

**Authors:** Timothy McKinnon, Rosemarie Venier, Brendan C. Dickson, Leah Kabaroff, Manon Alkema, Li Chen, Jack F. Shern, Marielle E. Yohe, Javed Khan, Rebecca A. Gladdy

**Affiliations:** ^1^ Lunenfeld-Tanenbaum Research Institute, Mount Sinai Hospital, Toronto, Canada; ^2^ Ontario Institute for Cancer Research, Cancer Stem Cell Program, Toronto, Canada; ^3^ Department of Surgery, University of Toronto, Toronto, Canada; ^4^ Department of Pathology & Laboratory Medicine, Mount Sinai Hospital, Toronto, Canada; ^5^ Genetics Branch, Oncogenomics Section, Center for Cancer Research, National Institute of Health, Gaithersburg, MD, USA

**Keywords:** sarcoma, rhabdomyosarcoma, mouse models of cancer, Kras, myogenic differentiation

## Abstract

While genomic studies have improved our ability to classify sarcomas, the molecular mechanisms involved in the formation and progression of many sarcoma subtypes are unknown. To better understand developmental origins and genetic drivers involved in rhabdomyosarcomagenesis, we describe a novel sarcoma model system employing primary murine p53-deficient myoblasts that were isolated and lentivirally transduced with Kras^G12D^. Myoblast cell lines were characterized and subjected to proliferation, anchorage-independent growth and differentiation assays to assess the effects of transgenic Kras^G12D^ expression. Kras^G12D^ overexpression transformed p53^−/−^ myoblasts as demonstrated by an increased anchorage-independent growth. Induction of differentiation in parental myoblasts resulted in activation of key myogenic regulators. In contrast, Kras-transduced myoblasts had impaired terminal differentiation. p53^−/−^ myoblasts transformed by Kras^G12D^ overexpression resulted in rapid, reproducible tumor formation following orthotopic injection into syngeneic host hindlimbs. Pathological analysis revealed high-grade sarcomas with myogenic differentiation based on the expression of muscle-specific markers, such as Myod1 and Myog. Gene expression patterns of murine sarcomas shared biological pathways with RMS gene sets as determined by gene set enrichment analysis (GSEA) and were 61% similar to human RMS as determined by metagene analysis. Thus, our novel model system is an effective means to model high-grade sarcomas along the RMS spectrum.

## INTRODUCTION

Soft tissue sarcomas (STS) are the most common extracranial solid tumors in childhood and rhabdomyosarcoma (RMS) is the most frequent STS in this population [1]. RMS likely arises from the skeletal muscle lineage as they can express muscle specific markers, MyoD1 (MYOD), Myogenin (MYOG) and desmin (DES). Thus, it has been suggested that transformation of early mesenchymal or skeletal muscle progenitors give rise to dysregulated skeletal muscle development in RMS [2].

RMS is categorized into distinct subtypes based on histopathology, including: embryonal (ERMS), alveolar (ARMS), pleomorphic (PRMS) and sclerosing/spindle cell (SRMS) RMS [3]. Genetic analyses of human RMS describe subtype-specific mutations that are used to define the biology and outcomes of the two main pediatric RMS subtypes, ERMS and ARMS [4–6]. ARMS typically contain fusion gene products of PAX3 or PAX7 and FOXO1 genes [5, 7] resulting from reciprocal balanced chromosomal translocations (t(2;13)(q35;q14) or t(1;13)(p36;q14)) [8]. In contrast, fusion-negative ERMS are more genetically heterogeneous and common genetic events in ERMS include: inactivation of the p53 pathway and activation of RAS signalling [9–12]. Recently, the genetic landscape of human RMS has identified possible oncogene or tumor suppressor gene combinations that may contribute to rhabdomyosarcoma initiation and progression [4]. Specifically, alterations in the receptor tyrosine kinase RTK/RAS/PIK3CA axis were the primary genetic signature in the majority of RMS samples.

Enhancing biologic models to validate candidate sarcoma genes will provide insight into genomic data and provide a platform for discovering new therapies. The majority of RMS animal models to date employ genetically engineered mice or transgenic zebrafish [13–15]. An emerging system to identify genes driving cancer formation is mosaic mice, which have successfully been used to model lymphoma and hepatocellular carcinoma [16–18]. Mosaic mouse models involve engraftment of host tissue with genetically manipulated progenitor cells and can be orthotopic and syngeneic. Thus, mosaic tumors develop in immunocompetent hosts with the appropriate microenvironment, which more faithfully recapitulates human disease. The use of tissue-specific progenitors as the cell of origin and the relative ease of genetic manipulation in donor cells allows for mosaic models to be employed in oncogenomic-based screens to functionally validate genetic data from human tumors.

In a recent study investigating the origins of RMS using developmentally restricted murine models, ERMS arose from Myf6-expressing, differentiating myoblasts with inactivated tumor suppressor genes, Trp53 or Ptch1 [19]. To determine whether cellular transformation of myoblasts can result in RMS, we created novel mosaic mouse models, which involved orthotopic injection and engraftment of p53^−/−^ myoblasts into syngeneic hosts. Oncogene delivery into myoblasts was performed using a lentiviral construct encoding Kras (Kras^G12D^) with a GFP reporter. Myoblasts were selected using fluorescence activated cell sorting (FACS) and injected into syngeneic mice, resulting in rapid formation of high-grade sarcomas with myogenic differentiation.

## RESULTS

### Characterization of p53-deficient myoblasts

To create mosaic sarcoma mouse models, skeletal muscle progenitors were isolated from p53-deficient mice as p53 is a known tumor suppressor in sarcoma and myoblasts are candidate cell of origin for RMS [19]. Parental myoblast cell lines generated from hindlimb skeletal muscle of neonatal p53^−/−^ pups (Myo25, Myo26) expressed muscle-specific RNA and protein and engrafted host muscle fibers following injection into neonatal p53^+/−^ pups (Supplementary Figure 1).

**Figure 1:**
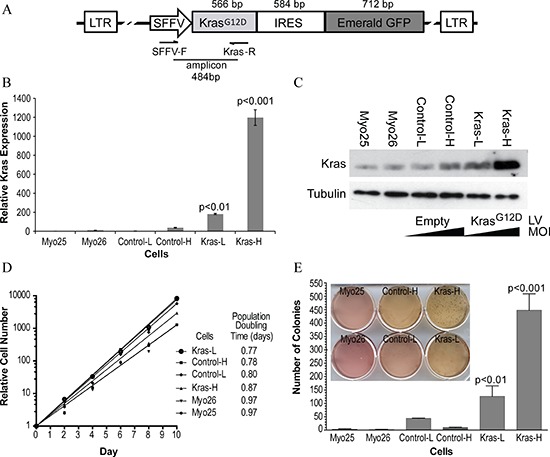
Effect of Kras^G12D^ overexpression on p53^−/−^ myoblast growth *in vitro* **A.** Schematic representation of the lentiviral expression vector construct. **B.** Quantification of relative proviral integrations in transduced cells was examined using real-time PCR. Numbers represent proviral copy number relative to parental cell lines (Myo25, Myo26). **C.** Cells transduced with Kras^G12D^ (Kras-L, Kras-H) demonstrate increased total Ras protein compared to empty vector (Control-L, Control-H) and parental cell lines as assessed by Western blot. LV: Lentivirus. MOI: multiplicity of infection. **D.** In transduced myoblast cell lines (Kras-H, Kras-L, Control-H, Control-L), there were no significant differences in proliferation assays between parental, empty vector or Kras^G12D^-overexpressing myoblasts. **E.** Kras^G12D^-overexpressing myoblasts cell lines demonstrate statistically significant (*p* < 0.001) increased colony formation in an anchorage-independent growth assay (inset). Error bars represent standard deviations.

Since oncogenic mutations of KRAS have been frequently described in RMS tumor samples, we employed constitutively activated Kras (Kras^G12D^) in our mosaic model. Lentiviral particles encoding bicistronic constructs of Kras^G12D^ and GFP were successfully delivered into the myoblast genome (Supplementary Figure 2-). To assess the effect of oncogene dosage, myoblasts were transduced with two different multiplicities of infection (MOI): a high MOI (i.e. 250) and a low MOI (i.e. 2). Control myoblasts were transduced with an empty vector encoding GFP alone at high and low MOIs. The following GFP-expressing p53^−/−^ myoblast lines were used: Kras-H (Myo25 + Kras^G12D^, high MOI), Control-H (Myo25 + empty vector, high MOI), Kras-L (Myo26 + Kras^G12D^, low MOI) and Control-L (Myo26 + empty vector, low MOI). Proviral integration was quantified with real-time PCR using primers specific to the viral promoter and Kras transgene (Figure 1A, B). Transgene integration was greater than 6-fold in Kras-H vs. Kras-L (*p* < 0.001) whereas empty vector myoblasts did not display significant proviral integration in the host genome. Increased proviral integration resulted in increased Kras protein levels in myoblasts as demonstrated by immunoblot (Figure 1C).

### Kras^G12D^ overexpression transforms p53^−/−^ myoblasts *in vitro*

To determine whether Kras^G12D^ overexpression in p53^−/−^ myoblasts resulted in cellular transformation, myoblast cell lines were characterized with proliferation, anchorage-independent growth and differentiation assays. Ten-day proliferation assays did not reveal significantly different rates of proliferation when comparing parental, empty vector control or Kras^G12D^ overexpressing myoblast cell lines (Figure 1D). Parental cell lines (Myo25, Myo26) underwent population doublings daily while cells overexpressing Kras^G12D^ had a slight reduction in doubling times, which was not statistically significant.

Since anchorage-independent growth is a hallmark of cellular transformation, myoblast cell lines were assayed (Figure 1E) and increased colony numbers were seen in Kras^G12D^-overexpressing myoblasts when compared to parental and empty vector controls. Furthermore, as Kras levels increased, so did colony numbers with Kras-H forming 4-fold more colonies than Kras-L (*p* < 0.001).

### Kras^G12D^ transduced myoblasts induce rapid tumor development

**Figure 2:**
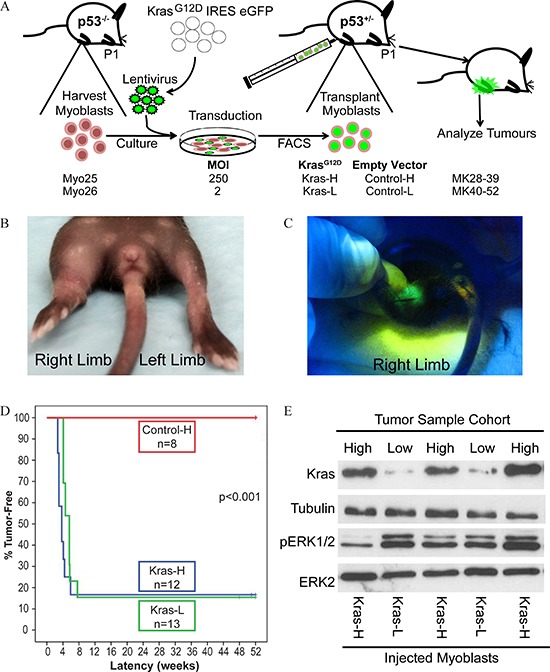
Injection of Kras^G12D^ expressing myoblasts into syngeneic host pups results in rapid tumor formation **A.** Schematic representation of mosaic mouse model involving injection of transduced p53^−/−^ myoblasts expressing activated Kras into postnatal day 1 (P1) host p53^+/−^ pups and assessed for tumor formation. **B.** Tumors formed rapidly at 3–6 weeks following injection of Kras^G12D^ -overexpressing myoblasts into host pup hindlimbs. **C.** Tumors were GFP^+^. **D.** Kaplan-Meier survival curves of mice injected with myoblasts transduced with Kras^G12D^ (Kras-L, Kras-H) and empty vector (Control-H) constructs. No tumors were found in control, empty vector alone injected cohorts aged up to one year post-injection. **E.** Western blot analysis of tumors demonstrates Ras expression in tumors from high and low Kras^G12D^ cohorts.

To determine if transformation of p53^−/−^ myoblasts with activated Kras, translated into tumor-forming capacity *in vivo*, myoblast cell lines were injected orthotopically into syngeneic hosts (Figure 2A). Myoblast cell lines, Kras-H or Kras-L, were injected intra-muscular (i.m.) into the right hindlimb of neonatal p53^+/−^ hosts; contralateral limbs were injected with myoblasts expressing the corresponding empty vector control constructs, Control-H or Control-L. Rapid GFP^+^ tumor formation was observed around weaning age in the right hindlimb of pups injected with Kras^G12D^ overexpressing myoblasts (Figure 2B, 2C). Mice injected with Kras-H had a median tumor latency of 3.4 weeks (range 3–6 weeks) with 83% penetrance (10/12) whereas mice injected with Kras-L had a median tumor latency of 4.6 weeks (range 4–6 weeks) with 85% penetrance (11/13, Table 1). Survival curves illustrate rapid tumor formation (Figure 2D) however no statistically significant survival differences were observed between high (Kras-H) versus low (Kras-L) Kras^G12D^ expressing mice demonstrating that increased Kras^G12D^ protein levels did not impact tumor latency. Metastatic disease was not observed following gross necroscopy in all experimental mice. Additionally, tumor formation did not occur in contralateral limbs injected with empty vector control myoblasts and observed for 52 weeks.

**Table 1:** Penetrance and latency of sarcoma formation in mosaic mice cohorts

Injected Cells					Host Mice	Tumors	
Myoblast Cell Line	Genotype	Transgene		MOI	Genotype	Penetrance (n)	Mean Latency (Range)
		Right Limb	Left Limb				
Myo25	p53^−/−^	Kras^G12D^	Empty Vector	High (250)	p53^+/−^	83% (10/12)	3.4 weeks (3–6 weeks)
Myo26	p53^−/−^	Kras^G12D^	Empty Vector	Low (2)	p53^+/−^	85% (11/13)	4.9 weeks (4–6 weeks)
Myo25	p53^−/−^	-	Empty Vector	High (250)	p53^+/−^	0 (0/8)	- (1 year post injection)

Host mice were injected with myoblasts overexpressing Kras^G12D^ and/or empty vector control myoblasts and followed for tumor formation for up to 52 weeks.

Immunoblots demonstrated that tumors from mice injected with Kras-H myoblasts had higher Kras levels than in tumors resulting from injection of Kras-L cells (Figure 2E). Since Erk1/2 are downstream of Ras in the mitogen-activated protein kinase (MAPK) pathway and are phosphorylated following Ras activation, we next examined Erk phosphorylation status to determine if increased Ras protein expression translated into MAPK pathway activation in mosaic mouse tumors. Although phosphorylated Erk1/2 was detected, phosphorylation levels did not correlate with the increased Ras protein levels found in tumors derived from injection of Kras-H myoblasts (Figure 2E).

### Pathologic analysis reveals high-grade sarcomas with myogenic differentiation

Tumors were assessed using WHO Classification [3]. Grossly, they were centered in skeletal muscle and circumscribed with variable extension into the surrounding muscle and subcutis. Tumor morphology was examined using hematoxylin and eosin (H&E) staining. The majority of tumors were spindle-epithelioid with a loose fascicular pattern with abundant and eosinophilic cytoplasm and patchy necrosis. Nuclei were ovoid, hyperchromatic and moderately pleomorphic; most tumors contained brisk mitotic activity (>20 per 1.7 mm^2^). Enlarged cells with rhabdoid morphology were occasionally observed within some tumors. Morphologically tumors were compatible with high-grade sarcomas.

**Figure 3:**
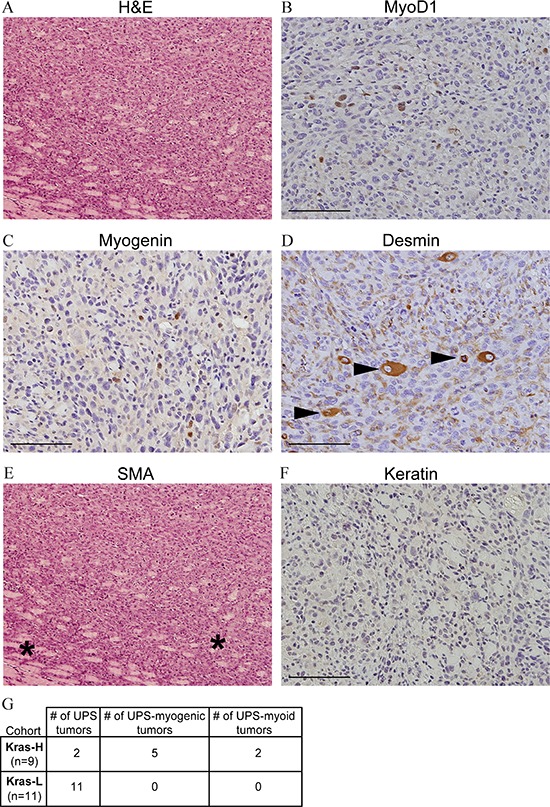
Generation of high-grade sarcomas with myogenic differentiation using mosaicism **A.** H&E staining of a representative tumor (100× magnification). To define if the tumors were sarcomas and to subtype all tumors, samples were subjected to immunohistochemical labelling of: Myod1 **B.** Myog **C.** Desmin **D.** SMA **E.** and Keratin **F.** Arrowheads in (D) show entrapped skeletal muscle and asterisks in (E) illustrate tumor vasculature. Bars represent 100 μm (B-E 200× magnification). **G.** Summary of histopathological analysis for Kras-H and Kras-L tumors.

A standard immunohistochemical panel was employed to classify the sarcomas. All tumors were negative for S100 and only one tumor was found to have rare immunoreactivity for pan-cytokeratin, thereby largely excluding neural crest and epithelial derivations, respectively. Five of 21 tumors had limited expression of one or more muscle specific transcription factors (Myog and/or Myod1), suggesting rhabdomyoblastic differentiation (Figure 3, Supplementary Table 1). Expression of myogenic markers was tumor-specific as it was remote from entrapped endogenous skeletal muscle; additionally, positive cells lacked multinucleation typical of atrophic and/or regenerating native skeletal muscle. Two additional tumors were immunoreactive for smooth muscle actin (SMA), indicating some degree of myoid differentiation (Supplementary Table 1). The remaining thirteen tumors were negative for myofibroblastic/muscle markers, suggesting an undifferentiated phenotype. As these high-grade sarcomas lacked a predominant line of differentiation, they are most analogous to undifferentiated pleomorphic sarcomas (UPS) in humans. There is currently no established threshold for the percentage of positive skeletal muscle markers required for RMS diagnosis; consequently, tumors bearing these markers are more appropriately classified as high-grade sarcoma with myogenic differentiation.

### Mosaic mouse sarcomas are enriched for human sarcoma gene expression patterns

**Figure 4:**
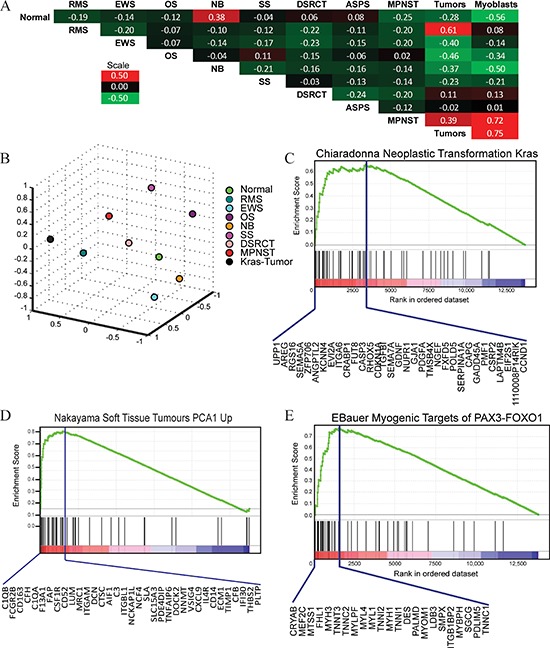
Metagene analysis and gene set enrichment analysis of mosaic mouse tumors **A.** Correlation between metagenes for normal human tissue, pediatric cancers, and Kras-expressing mosaic tumors. Each category is represented by the mean expression value among the samples that comprise the group. Similarity among the metagenes was determined by the Pearson's correlation coefficient between the mean values. RMS - rhabdomyosarcoma, EWS - Ewing sarcoma, OS - osteosarcoma, NB - neuroblastoma, SS - synovial sarcoma, DSRCT - desmoplastic small round cell tumor, ASPS - alveolar soft part sarcoma, MPNST - malignant peripheral nerve sheath tumor. Kras-tumor represents four mosaic tumors. **B.** Multi-dimensional scaling plot demonstrating the distance between the metagenes for normal human tissue, pediatric cancers and Kras-expressing tumors. **C.** GSEA analysis of Kras-expressing myoblasts demonstrates gene set enrichment with signature of oncogenic Kras transformation. Enrichment score is 0.65, *p* < 0.01. Genes comprising the leading edge are delineated below the enrichment plot. **D.** GSEA analysis of tumor samples demonstrated gene set enrichment with a UPS signature (ES = 0.82, *p* < 0.001) and **E.** myogenic targets of PAX3-FOXO1 (ES = 0.77, *p* < 0.01).

To investigate if our mosaic mouse tumors were consistent with human sarcoma gene expression patterns, mRNA from myoblasts and tumor tissue was examined with gene expression microarrays. Metagene analysis was performed to determine the correlation between mosaic mouse sarcomas and human pediatric tumors [19]. The mosaic mouse tumors most significantly correlated with human RMS (61% similar, Figure 4A, 4B) and were also 39% similar to human malignant peripheral nerve sheath tumor (MPNST), a sarcoma known to be driven by oncogenic RAS. Gene set enrichment analysis (GSEA) was performed to investigate whether mosaic mouse sarcomas were enriched in human tumor gene signatures (Figure 4C–E) [20]. Since myoblasts were transformed by oncogenic Kras, it was not surprising to find enrichment for a gene set specific to neoplastic transformation with Kras (Enrichment score (ES) = 0.65, *p* < 0.01, Figure 4C). The leading edge of this enrichment contains genes whose expression is induced by activated Kras including cyclinD1 (CCND1). Kras-expressing tumors were also enriched for gene sets specific to undifferentiated pleomorphic sarcomas (ES = 0.82, *p* < 0.001, Figure 4D) and myogenic targets of the ARMS specific transcription factor PAX3-FOXO1 (ES = 0.77, *p* < 0.01, Figure 4E). The leading edge of this enrichment includes several markers of myogenic differentiation including desmin (DES), several myosins (MYH1, MYH3, MYL1, MYL4) and several troponins (TNNC1, TNNC2, TNNI1, TNNI2, TNNT3). Together, these results confirm oncogenic Kras expression as the transformative event in our mosaic mouse tumors and substantiate histopathological results, which classified the tumors as high-grade sarcomas with myogenic differentiation.

### Impaired myogenic differentiation in Kras^G12D^ transformed p53^−/−^ myoblasts

**Figure 5:**
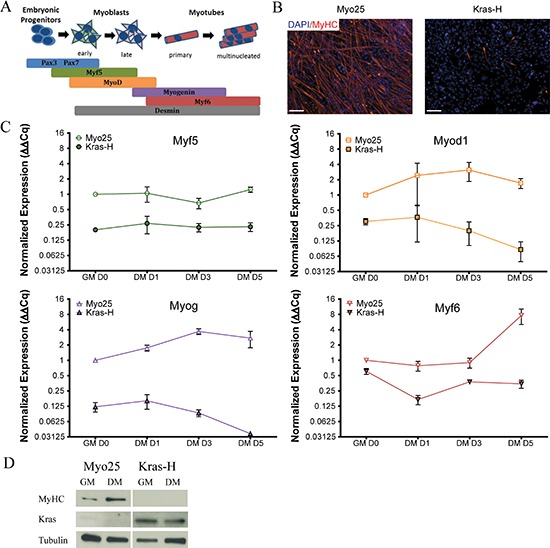
Kras transduced myoblasts fail to differentiate *in vitro* **A.** Sequential activation of myogenic transcription factors facilitates differentiation of myogenic precursors to mature muscle fibers. **B.** Parental myoblasts (Myo25) differentiated as illustrated by extensive MyHC immunolabel after switching from high serum growth medium (GM) to 5 days in low serum differentiation medium (DM) whereas Kras^G12D^ overexpressing myoblasts (Kras-H) failed to differentiate. Scale bar represents 100 μm. **C.** Expression of the myogenic regulatory factors Myf5, Myod1, Myog and Myf6 in Kras^G12D^ overexpressing myoblasts did not follow expression trends observed in parental myoblasts. **D.** Immunoblot illustrates that Kras-transformed myoblasts (Kras-H) did not express MyHC *in vitro* after 5 days in differentiation medium in contrast to parental cell lines (Myo25).

Since we did not generate classic RMS in our model, we next examined if Kras overexpression affected skeletal muscle differentiation (Figure 5). Following five day incubation in differentiation media (DM) [21], myoblasts were assayed for Myosin Heavy Chain (MyHC) expression, a structural protein in mature muscle fibers. The parental p53^−/−^ cell line Myo25 terminally differentiated as seen by the presence of a large number of multinuclear, MyHC^+^ muscle fibers (Figure 5B). In contrast, transduced cells overexpressing Kras^G12D^ had minimal numbers of elongated multinuclear MyHC^+^ cells.

In parallel, gene expression profiles of muscle-specific markers were examined to determine whether the decrease in the number of MyHC^+^ muscle fibers occurred concomitantly with alterations in myogenic gene expression levels during differentiation (Figure 5C). Gene expression levels in Myo25 and Kras-H myoblasts were expressed over the course of the experiment relative to expression levels in undifferentiated parental cells at Day 0 (Figure 5C). As expected, expression levels in the parental Myo25 cell lines followed expected differentiation patterns as terminally differentiated muscle fibers formed. In contrast, muscle-specific gene expression levels in Kras^G12D^ overexpressing Kras-L were decreased before the induction of differentiation demonstrating a low level expression of these markers. Expression of myogenic markers in Kras-H were either unchanged (Myf5) or decreased (Myf6) after 5 days in DM, demonstrating impaired differentiation with Kras^G12D^ overexpression. Finally, myoblast protein lysates were subjected to immunoblot analysis to examine expression of the mature muscle fiber marker MyHC (Figure 5D) and Myo25 and Kras-H lysates were simultaneously probed with a Kras-specific antibody. As expected, MyHC protein levels increased in parental Myo25 cells following the 5 day differentiation assay, however Kras-H cells with high Kras protein levels did not express MyHC at the onset of the differentiation assay or following 5 day incubation in DM further indicating impairment of skeletal muscle differentiation.

## DISCUSSION

### Mosaic mice as a functional genomics tool for modelling sarcomas

Despite recent reports in defining the genetic landscape of pediatric sarcomas, survival rates for patients in high-risk categories have not changed in the past decade as it is still unknown which molecular events are key for sarcomagenesis [1]. Thus, it is imperative that better methods for modeling the biology of these devastating tumors are developed. In this study, we describe a novel murine model of sarcoma using mosaicism. Tumors were generated in syngeneic mice following orthotopic engraftment of donor cells into the skeletal muscle of immunocompetent hosts. To effectively model human disease, mutations described in patient tumors (i.e. p53 loss-of-function, oncogenic Kras) were selected and the differentiating myoblast, the putative cell of origin for RMS was studied. High-grade sarcomas arose 3–6 weeks following myoblast engraftment and contained regions of myogenic differentiation although the majority of the tumor consisted of undifferentiated mesenchymal cells. Gene expression analysis corroborated histopathological analysis as gene expression patterns of mosaic mouse tumors were highly enriched for human sarcoma gene signatures. Thus, the use of mosaic mice has several advantages for investigating biologic events involved in sarcomagenesis as these models can be rapidly generated and easily adapted as new genetic data and experimental techniques emerge.

### Genetic drivers and developmental origins of rhabdomyosarcomas

We report that p53^−/−^ myoblasts overexpressing Kras^G12D^ formed high-grade sarcomas with myogenic differentiation. Although it was initially hypothesized that syngeneic engraftment of transformed myoblasts would result in RMS, there is evidence to suggest that RMS represent a spectrum of tumors with varying levels of myogenic differentiation, which may explain the phenotype observed [19]. Indeed, even amongst human studies of rhabdomyosarcoma enriched for the embryonal subtype, there exists conspicuous heterogeneity in the extent of myogenin expression [22]. The degree of tumor differentiation along this continuum is likely affected by the transforming mutation(s) introduced and/or the cell of origin that the tumors arise from. Thus, our mosaic models provide an ideal system to investigate the interplay between oncogenic drivers and how the developmental context in which they arise impact on RMS biology.

Kras activation and p53 deficiency have been described in human sarcomas and genetically engineered animal models have also been generated using this combination [9–12]. While the majority of murine tumors analyzed were described as RMS, most previous studies were limited, as they did not directly assess the extent of myogenic differentiation. For example, the presence of MyoD1^+^ cells in murine tumors was deemed sufficient for RMS classification, as MyoD1^+^ rhabdomyoblasts are diagnostic for human RMS. However, since our tumors developed orthotopically in host skeletal muscle, care was exercised to ensure that reactive/atrophic MyoD1^+^ entrapped skeletal muscle cells were distinguished from MyoD1^+^ tumor cells. Mosaic mouse tumors were mostly undifferentiated despite the presence of patchy/focal MyoD1 protein expression and thus could not be irrefutably classified as RMS. Interestingly, histopathological differences were noted between tumors arising following injection of either Kras-H or Kras-L myoblasts. Whether these differences resulted from increased Kras expression levels in Kras-H myoblasts or represented intrinsic differences between the parental myoblasts (i.e. Myo25 vs. Myo26) has yet to be determined. Real-time PCR profiling substantiated the histopathology, as the levels of myogenic markers were lower in tumors compared to normal skeletal muscle controls (Supplementary Figure 4). Additionally, metagene analysis demonstrated that expression profiles of mosaic mouse tumors were 61% similar to human RMS (Figure 4A).

To determine if the undifferentiated tumor phenotype arose in cells that had impaired myogenic differentiation, we assessed the capacity of myoblasts to terminally differentiate into skeletal muscle. In this study, transgene expression was driven by a heterologous promoter and resulted in Kras^G12D^ overexpression, which is a limitation of lentiviral transduction. In future investigations, genome editing may be used to circumvent this limitation, allowing for the expression of mutant constructs under the control of endogenous genetic elements. Transduced p53^−/−^ myoblasts overexpressing Kras^G12D^ were unable to differentiate as assayed by expression of MyHC a marker of terminally differentiated muscle fibers. In contrast, parental cell lines retained the capacity to differentiate and formed MyHC^+^ myotubes. Both p53 inactivation and Ras overexpression have been previously implicated in myoblast differentiation [23–25]. However in our study, loss of p53 did not appear to be responsible for the inhibition of differentiation as parental cells (i.e. Myo25) retained the capacity to differentiate. Future studies will attempt to clarify the role of this genetic combination on differentiation in mosaic mouse sarcoma models.

The cellular origin of RMS has long been debated. As RMS can develop in multiple anatomical sites, it is plausible that different cellular lineages contribute to RMS development. Overall, myoblasts remain the most vetted cell of origin for RMS. This concept was initially demonstrated when human skeletal muscle progenitors were transformed using SV40 T/t antigen, human telomerase reverse transcriptase (TERT) and Hras^V12G^ which resulted in RMS formation in murine xenografts [26]. When homozygous deletion of p53 was lineage-restricted in different muscle cell populations, although ERMS predominately arose from the maturing Myf6^+^ myoblast lineage, other skeletal muscle progenitors could give rise to different tumors on the RMS spectrum following loss of p53 [19]. In contrast, in a recent report RMS arose following transformation of Pax7^+^ myogenic progenitors with the same mutations while transformation of Myod^+^ progenitors resulted in UPS [27], illustrating how cellular lineage can affect sarcomagenesis. When oncogenic Kras^G12V^ was expressed in p16/p19^null^ satellite cells pleomorphic RMS developed following injection into adult, cardiotoxin injured skeletal muscle [28] which also supports the notion that RMS arise from different stem cell populations with varying degrees of differentiation. However, how this particular genetic combination affected the satellite cells' capacity to differentiate is not known. Finally, other mesenchymal lineages have been reported to give rise to RMS or RMS-like tumors as lineage-restricted activation of the Sonic hedgehog pathway in adipocytes, resulted in the formation of tumors that were classified as RMS [29]. The debate in defining the cell of origin in RMS may also be explained by the nature of the oncogenic mutations used to drive rhabdomyosarcomagenesis.

### Human sarcomas are effectively modeled by mosaic mice

In addition to providing biologic data on the process of cellular transformation, mouse models are the gold standard to model human disease. Gene expression analysis performed compared our murine tumors to the genetic signatures of different human sarcomas. Using GSEA, the gene expression signature of tumors that arose following engraftment of p53^−/−^ Kras^G12D^ overexpressing myoblasts was enriched for human UPS and ARMS gene signatures, illustrating some degree of skeletal muscle differentiation with our murine sarcomas. Importantly, mosaic mouse tumors generated were most similar to human RMS, as determined by metagene analysis. Therefore, murine tumors generated by overexpressing Kras^G12D^ in p53-deficient differentiating myoblasts, recapitulate human malignancies and are an effective means to model high-grade sarcomas along the RMS spectrum. As additional mosaic mouse models are generated using different oncogenic mutations, it will be essential to investigate how the transcriptome is altered and correlate this profile with what is observed in tumor phenotype.

In conclusion, the creation of mosaic mouse models effectively recapitulates human sarcoma formation and future work using this approach will help uncover the developmental context and the genetic alterations that are required for sarcomagenesis. To translate this critical knowledge into improved patient care, mosaic models are also powerful tools for testing novel therapeutic agents.

## MATERIALS AND METHODS

### Myoblast generation and characterization

Experiments were performed using C57Bl/6^p53tm1/Tyj^ mutant mice [30]. Animal protocols were in accordance with the Animal Care Committee at the Toronto Centre for Phenogenomics. Primary myoblast harvest and culture were performed as previously described [21]. Two p53^−/−^ mouse myoblast cell lines (Myo25, Myo26) were generated and propagated using Myoblast Growth Media (MGM, 50% F10/50% DMEM, 20% FBS, pen/strep, 25 ng/ml bFGF). Myoblasts expressed muscle-specific genes and proteins upon differentiation into functional muscle fibers *in vitro* (Supplementary Figure 1).

### Lentiviral transduction of myoblasts

Lentiviral constructs were produced using protocols from the RNAi Consortium (http://www.broadinstitute.org/scientific-community/science). Second generation packaging (pCMV-dR8.74psPAX2) and envelope (pMD2.G) plasmids and lentiviral expression vectors described below were transfected into 293T cells with Effectene^®^ Transfection Reagent (Qiagen, Mississauga). Murine Kras^G12D^ (GenBank: 16653) was subcloned between BamHI/XhoI restriction enzyme sites preceding IRES-Emerald (GFP) in the pHR-SIN-SFFV vector (Dr. Joao Goncalves, Toronto). Lentiviral particles in conditioned media from 293T cells at 24 and 48 h following cotransfection with either pHR-SIN-SFFV-Kras^G12D^-IRES-Emerald or pHR-SIN-SFFV-IRES-Emerald (empty vector) were pooled and centrifuged (1500 × g, 5 min), filtered (20 μm pore size) and stored at −80°C. Lentiviral titer was determined using a Lenti-X™ p24 Rapid Titer ELISA (Clontech, Mountain View, CA). MOI was calculated based on lentiviral titer. High (250) or low (2) MOIs were used and myoblasts were incubated with MGM containing lentiviral particles for 16 h, washed and cultured for 6 days. GFP^+^-expressing cells were isolated using FACS and expanded. Myoblast Kras^G12D^ transgene expression was confirmed using PCR and protein overexpression was confirmed with immunoblots (Supplementary Table 2). Myoblast proviral integration was quantified using transgene-specific primers and real-time PCR (Supplementary Table 2).

### *In vitro* assessment of transformation

Proliferation was examined by plating 2 × 10^5^ cells onto collagen-coated 10 cm tissue culture plates (Day 0). Cells were passaged every 2 days and counted (ViCell, Beckman Coulter). The ability of myoblasts to grow on soft agar was assessed using standard protocols [31].

### Transplantation of myoblasts in syngeneic hosts

Myoblast engraftment of syngeneic host skeletal muscle was performed as previously described [21]. Intra-muscular (i.m.) injections were performed using 5 μl cell supension along with < 1 μl Spot Endoscopic Marker (GI Supply, USA) in one-day old pup skeletal muscle. Myoblasts expressing Kras^G12D^ were injected into the right hindlimb, empty vector controls into the contralateral limb and monitored for tumor formation.

### Pathology

Mice were sacrificed when tumors measured 1.5 cm^3^ or other experimental end points were reached, including: respiratory compromise or evidence of malnourishment. Necropsy was performed, tumor and contralateral control limb skeletal muscle were harvested and underwent standard fixation. Formalin-fixed, paraffin-embedded tissue was cut into 4 μm sections for H&E staining and immunohistochemical analysis. Antibodies are described in Supplementary Table 2 and control stains displayed in Supplementary Figure 3. For mouse primary antibodies, a Mouse-on-Mouse Peroxidase kit (Vector Labs, Burlington, ON) was used. All sections were reviewed with a dedicated sarcoma pathologist (BCD) (Supplementary Table 1).

### Protein expression analysis

Proteins were harvested following incubation in RIPA Lysis Buffer including c0mplete Mini, EDTA-free protease (Roche) and phosphatase (Sigma) inhibitors, followed by centrifugation (4°C, 10000 × g, 15 min) and quantification of protein concentration using the DC™ Assay (Bio-Rad, Mississauga, Canada). Protein samples were separated and transferred to PVDF membranes using standard western blot conditions. Membranes were blocked and incubated in primary/secondary antibody solutions (Supplementary Table 2) for 1 h, RT (5% BSA) and washed (TBS-T, 3 × 10 min) between steps. Immunolabelled proteins were visualized utilizing ECL system (GE Life Sciences, Quebec).

### Gene expression microarray analysis

To evaluate global gene expression, RNA was harvested from cell or tumor lysates, was *in vitro* transcribed, hybridized and applied to Affymetrix Mouse 430A arrays (Affymetrix, Santa Clara, CA). For gene set enrichment analysis (GSEA), gene expression data were normalized to empty vector controls by standard methods and log_2_-transformed (Supplementary Table 3). The median of the transformed gene expression from myoblast cell lines stably expressing empty vector (Control-H, Control-L) was subtracted from the median of transformed gene expression from four tumors (MK33, MK36, MK40 and MK43) or from cell lines stably expressing Kras^G12D^ (Kras-H, Kras-L). Genes were ranked according to the absolute fold-change in expression (Supplementary Tables 4–8) using a Signal2Noise metric. GSEA analysis (http://www.broadinstitute.org/gsea/index.jsp) was performed using default parameter settings on two ranked lists. Published gene sets of rhabdomyosarcomas and other pediatric malignancies were used in this analysis [19, 32]. Significance was defined as having FDR *q*-value < 0.25 and FWER *p*-value of < 0.05. The metagene projection method [33] was used to determine similarity between gene expression profiles of the mosaic mouse tumors described above and gene expression profiles of pediatric solid tumors or normal controls (GEO, http://www.ncbi.nlm.nih.gov/gds/) which was performed as previously described [32].

### Assessment of myoblast *in vitro* differentiation

Myoblast differentiation into muscle fibers was examined using standard conditions [21]. Myoblasts were seeded onto collagen-coated coverslips and differentiation was induced by replacing growth media with differentiation media (DMEM, 5% horse serum, pen/strep) for 5 days then cells were fixed with 4% PFA for 10 min, immunolabeled with myosin heavy chain (MyHC, Clone MF20) antibody and counterstained with DAPI. Cells were imaged using an Olympus 1X51 fluorescence microscope.

### Proviral integration PCR

To evaluate proviral integration into the myoblast genome and transgene representation in tumor tissue, PCR was performed on genomic DNA using primers and conditions detailed in Supplementary Table 2. Real-time PCR was performed using mouse cell and tissue samples to quantify gene expression levels are described in Supplementary Methods and primer sequences are listed in Supplementary Table 2.

## SUPPLEMENTARY DATA FIGURES AND TABLES




