# Supplementary material for: Kras activation in p53-deficient myoblasts results in high-grade sarcoma formation with impaired myogenic differentiation
Source: Oncotarget. 2015 May 15;6(16):14220–32. doi: 10.18632/oncotarget.3856 (PMC4546462; doi:10.18632/oncotarget.3856)
Supplement: Supplementary file 1 [file oncotarget-06-14220-s001.pdf]

SUPPLEMENTARY DATA

Characterization of p53-deficient myoblast lines

Myoblasts were harvested from the hindlimb skeletal muscle of neonatal $p53^{-/-}$ limb and cultivated *in vitro* according to protocols previously described (21). After approximately 10 passages, during which, fibroblasts were depleted, primary $p53^{-/-}$ myoblasts cell lines survived. These cultures (Myo25, Myo26) demonstrated a skeletal muscle lineage due to the expression of muscle-specific RNA and protein (Supplementary Figure 1). Additionally, both Myo25 and Myo26 form muscle fibers following *in vitro* differentiation protocols. These cells maintained *in vivo* functionality as illustrated by their engraftment into syngeneic host skeletal muscle following injection into neonatal $p53^{+/-}$ host pups (Supplementary Figure 1).

Assessment of proviral integration in mouse tumor tissue

Proviral representation in harvested mouse tissues was examined using PCR. Primers specific to the transgene exogenous promoter (SFFV) and $Kras^{G12D}$ sequence (Figure 1) were utilized (Supplementary Table 2). Tumor or control limb tissues were harvested and flash frozen in liquid nitrogen at endpoint. DNA extraction from pooled tissue involved cold pulverization with mortar and pestle followed by DNA isolation using kit protocols (DNeasy Blood and Tissue Kit, Qiagen). Genomic DNA was then subjected to PCR amplification (30 cycles) using the described primers. PCR products were visualized following agarose electrophoresis using AlphaImager® EP (Alpha Innotech) and Alpha Imager software v1.2.0.1. Amplification of GAPDH from all tissues demonstrated presence of sufficient DNA.

p53 genotyping

p53 genotyping PCR were performed for all experimental animals and primary myoblast cell lines as previously described (30). DNA was isolated from samples using DNeasy Kit (Qiagen). Multiplex PCR was performed using approximately 100 ng genomic DNA. PCR mix and cycling parameters strictly followed protocols described by the Jackson Laboratory (jaxmice.jax.org/protocolsdb/f?p=116:2:386824144988 6939::NO:2:P2_MASTER_PROTOCOL_ID,P2_JRS_ CODE:5741,002101).

Quantitative real-time PCR (qPCR)

For gene expression analysis, qPCR was used to determine expression levels in mouse cell lines and tumor tissue. RNA was harvested from parental and transduced myoblasts following on-plate lysis and homogenization according to manufacturer's instructions (QIAShredder with RNeasy kit, Qiagen). Tumor tissue was excised from mice and snap-frozen in liquid-nitrogen before being stored at -80°C until required. Tissue was processed less than 6 months after excision. RNA was harvested from mouse tumor tissue following pulverization in liquid nitrogen using a mortar and pestle. Tissue lysis and homogenization was performed using QIAShredder columns (Qiagen) and RNA isolated with RNeasy kit (Qiagen). For all samples, on-column DNA digestion was performed according to kit protocols. RNA concentration and purity was measured on the NanoDrop8000 (Thermo Scientific). First-strand synthesis was performed with 2 µg total RNA in 40 µl total volume using qScript cDNA Synthesis Kit according to the described protocols on a C1000™ Thermal Cycler (BioRad). Gene-specific, real-time PCR was performed using Power SYBR (Applied Biosystems) on the CFX384 Real-time PCR cycler (BioRad) according to manufacturer's description. Primer specificity was tested *in silico* using BLAST.

Statistical analysis

Statistical analyses of *in vitro* experiments were performed using the GraphPad Prism 5 Software. Proviral integration PCR was performed in technical triplicate; biological triplicate experiments were combined and analyzed using one-way analysis of variance (ANOVA) followed by Tukey's multiple comparison test. Technical triplicates were quantified for proliferation assays and analyzed with one-way ANOVA and Tukey's multiple comparison test. Anchorage-independent growth assays were compiled from biological triplicates and analyzed by one-way ANOVA and Tukey's multiple comparison test. Survival curves were calculated using the Kaplan-Meier method and were generated using SPSS (IBM, version 19).

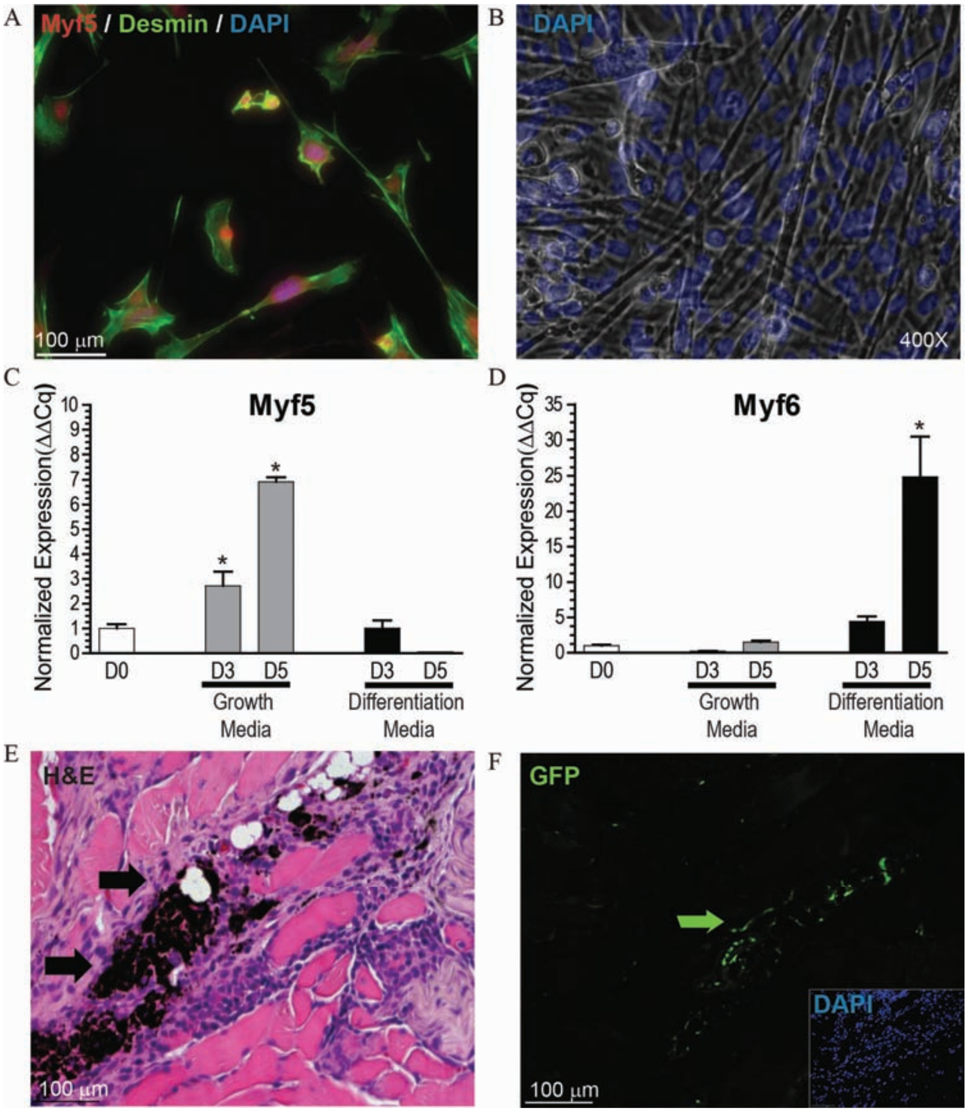

Supplementary Figure S1: Characterization of myoblasts based on expression of muscle-specific developmental markers and engraftment into syngeneic host skeletal muscle tissue. Immunocytochemistry demonstrates that freshly-harvested myoblast cultures express known muscle markers, such as Myf5 and desmin **A.** p53-deficient myoblasts fuse into syncytial muscle fibers *in vitro* following 7 days in differentiation media (DM). Brightfield image with an overlay of fluorescence micrograph of nuclei counterstained with DAPI **B.** Real-time PCR demonstrates established myoblast cultures express Myf5 under standard growth conditions **C.** Upon induction of differentiation, myoblasts downregulate Myf5 RNA levels while concomitantly upregulating Myf6. Expression levels at Day 3 (D3) and Day 5 (D5) after induction of differentiation are shown relative to myoblast expression levels at Day 0 (D0) in standard growth conditions **D.** Statistical significance was tested using 1-way ANOVA with Tukey post-test to compare gene expression levels to gene expression at D0 (*$p < 0.05$). p53 mutant myoblasts engraft syngeneic host hindlimbs as shown with H&E staining and GFP IHC on serial sections of muscle tissue at 28 days after myoblast injection **E.** Injection dye marks the site of injection (black arrows) where GFP^{+} cells **F.** (green arrow) can also be found.

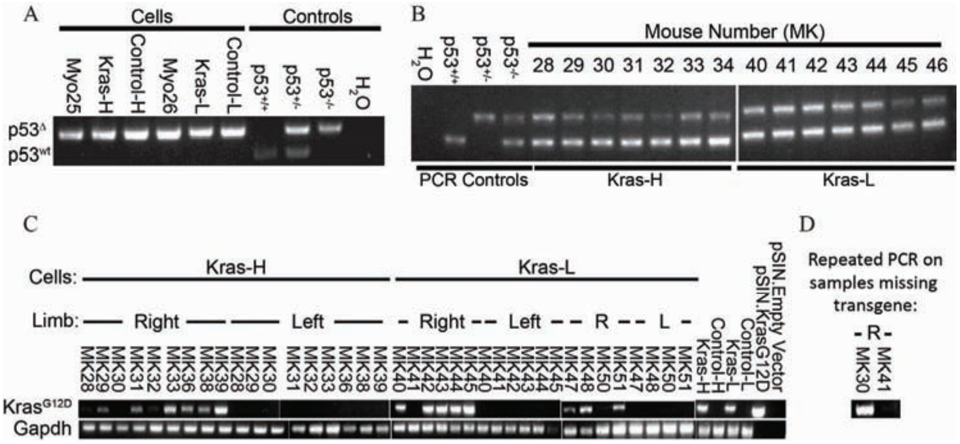

Supplementary Figure S2: Genotyping of donor cells demonstrates that the injected cells are p53 homozygous null ($p53^{-/-}$). A. Genotyping of host mice reveals that mice are p53 heterozygotes ($p53^{+/-}$) **B.** PCR with viral promoter forward primer and $Kras^{G12D}$ reverse primer reveal that all tumor samples harvested from right limbs injected with transformed myoblasts contain $Kras^{G12D}$ transgene **C.** Tissue harvested from left limbs injected with empty vector control myoblasts did not contain transgene. PCR did not amplify the specific amplicon in two samples (MK30, MK41) in the first round of PCR. A second PCR reaction with increased tumour DNA demonstrated that samples contained transgene **D.**

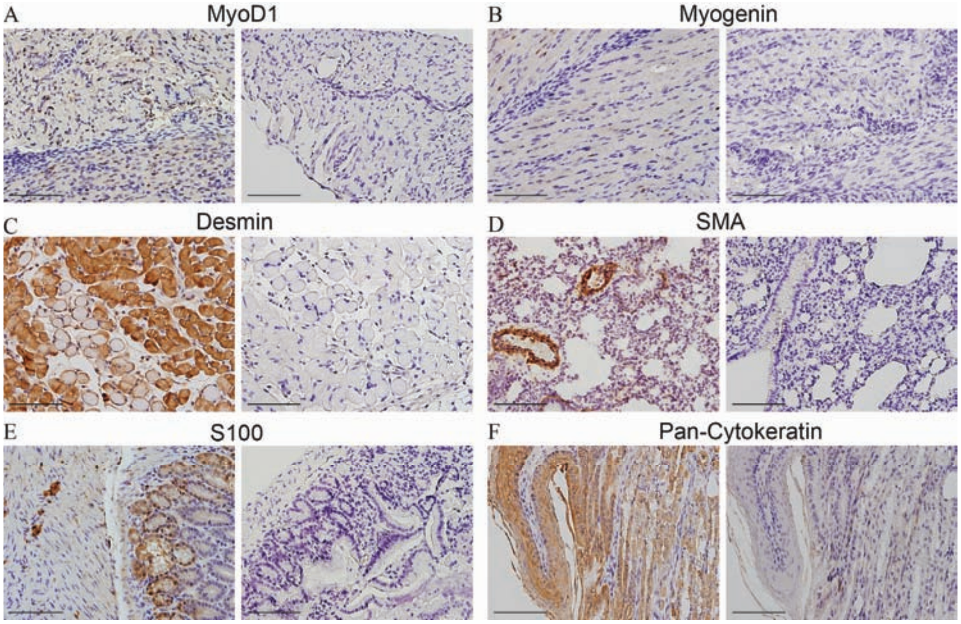

Supplementary Figure S3: Control immunohistochemistry for pathological assessment of murine tumors. Neonatal mouse skeletal muscle was immunolabeled with antibodies against MyoD1 **A.** or Myogenin **B.** Antibodies against Desmin were used to probe adult mouse skeletal muscle **C.** SMA-specific antibodies were used to label adult mouse lung tissue **D.** 200X magnification (A–D). Adult mouse duodenum **E.** was immunolabeled with anti-S100 antibodies and adult stomach **F.** with pan-cytokeratin. 100X magnification (E) and 40X magnification (F) In all experiments, probing with the appropriate secondary antibody alone (2° alone) was used a negative control.

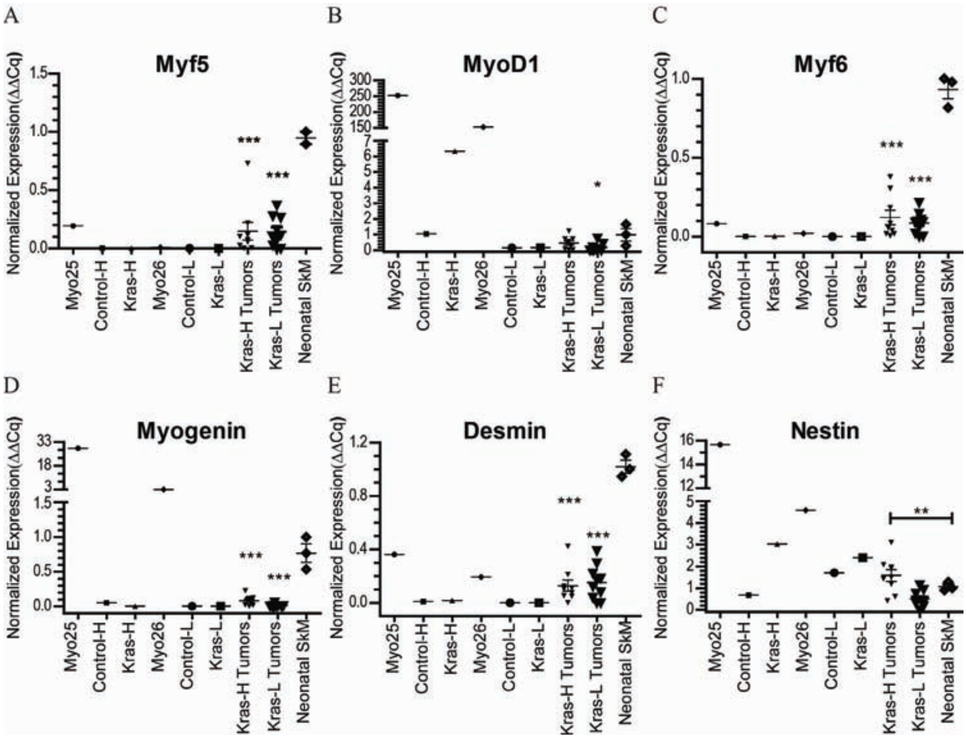

Supplementary Figure S4: Skeletal muscle markers are reduced in Kras-driven tumors compared to normal skeletal muscle tissue. Expression of genes involved in skeletal muscle development was quantified with quantitative PCR using gene specific primers and expression normalized to Gapdh and β-actin expression. Data shown here is relative to gene expression levels in normal neonatal mouse skeletal muscle samples (*n* = 3). Statistical significance between tumor cohorts and control muscle tissue was calculated using one-way ANOVA and pairwise comparison with Bonferonni's multiple comparison test (**p* < 0.05, ***p* < 0.01, ****p* < 0.001).

Supplementary Table S1. Detailed results from histopathological assessment of murine tumors following immunohistochemistry

Donor Cells	Case	MyoD1	Myogenin	Desmin	SMA	S100	PanCK	Impression
Kras-H	MK28	2	1	0	blush	0	0	High-grade sarcoma with rhabdomyoblastic differentiation
	MK29	1	0	0	blush	0	0	High-grade sarcoma with rhabdomyoblastic differentiation
	MK31	1	1	1	2	0	0	High-grade sarcoma with rhabdomyoblastic differentiation
	MK32	0	0	0	1	0	0	High-grade sarcoma with myoid differentiation
	MK33	3	1	1	0	X	0	High-grade sarcoma with rhabdomyoblastic differentiation
	MK34	0	0	0	1	0	0	High-grade sarcoma with myoid differentiation
	MK36	0	0	0	0	0	0	High-grade sarcoma, undifferentiated
	MK38	0	0	0	0	0	0	High-grade sarcoma, undifferentiated
	MK39	1	0	0	1	0	0	High-grade sarcoma with rhabdomyoblastic differentiation
Kras-L	MK40	0	0	0	0	0	1	High-grade sarcoma, undifferentiated*
	MK41	0	0	0	0	0	0	High-grade sarcoma, undifferentiated
	MK42	0	0	0	0	0	0	High-grade sarcoma, undifferentiated
	MK43	0	0	0	0	0	0	High-grade sarcoma, undifferentiated
	MK44	0	0	0	0	0	0	High-grade sarcoma, undifferentiated
	MK45	0	0	0	0	0	0	High-grade sarcoma, undifferentiated
	MK47	0	0	0	0	0	0	High-grade sarcoma, undifferentiated
	MK48	0	0	0	0	0	0	High-grade sarcoma, undifferentiated
	MK49	0	0	0	0	0	0	High-grade sarcoma, undifferentiated
	MK50	0	0	0	0	0	0	High-grade sarcoma, undifferentiated
	MK51	0	0	0	0	0	0	High-grade sarcoma, undifferentiated

*presume keratin is aberrant, morphology similar to other tumours

Score	% Positive
0	none
1	<1%
2	1-10%
3	10-33%
4	22-66%
5	>66%

Supplementary Table S2. Source and use of antibodies and primers

**Primary Antibodies:**			
**Antigen (acronym) [clone]**	**Source**	**Host**	**Dilution (Usage[a])**
α-tubulin [DM1A]	Sigma	Mouse	1:5000 (IB)
Desmin [D33]	Dako	Mouse	1:50 (IHC)
Desmin [DEU-10]	Abcam	Mouse	1:200 (ICC)
Fibroblast growth factor receptor 4 (FGFR4) [C16]	Santa Cruz Biotechnologies	Rabbit	1:1000 (IB)
Green Fluorescent Protein (GFP)	Molecular Probes	Rabbit	1:1000(IHC)
Keratin [AE3]	Millipore	Rabbit	1:50 (IHC)
Myf5 [C-20]	Santa Cruz Biotechnologies	Rabbit	1:100 (ICC)
MyoD1 [5.8A]	Dako	Rabbit	1:100 (IHC)
Myogenin [F5D]	Becton Dickinson	Rabbit	1:100 (IHC)
Myosin Heavy Chain (MyHC) [MF20]	R&D Systems	Mouse	1:200 (ICC)
Ras [F234]	Santa Cruz Biotechnologies	Rabbit	1:500 (IB)
S100A4	Dako	Rabbit	1:200 (IHC)
Smooth muscle actin (SMA) [1A4]	Dako	Mouse	1:200 (IHC)
**Isotype Control Antibodies:**			
Mouse IgG	Santa Cruz Biotechnologies	Mouse	[b] (ICC)
Rabbit IgG	Santa Cruz Biotechnologies	Mouse	[b] (ICC)
**Secondary Antibodies:**			
Anti-mouse IgG-AlexaFluor488	Molecular Probes	Donkey	1:500 (ICC)
Anti-mouse IgG-AlexaFluor568	Molecular Probes	Donkey	1:500 (ICC)

(Continued)

Anti-rabbit IgG-AlexaFluor488		Molecular Probes	Donkey	1:500 (ICC)
Anti-rabbit IgG-AlexaFluor568		Molecular Probes	Donkey	1:500 (ICC)
Anti-mouse IgG-HRP		GE Healthcare	Sheep	1:5000 (IB)
Anti-rabbit IgG-HRP		Santa Cruz Biotechnologies	Goat	1:5000 (IB)
**Real-Time PCR Primers:**				
**Gene Symbol(Accession Number)**	**Primer Sequence (5′→3′)**	**T_a (°C)[c]**	**Amplicon (bp)**	**Source**
Myod1(NM_010866)	F:CCCGGCGGCAGAATGGCTAC<br>R:GGAGTGCCTACGGTGGTGCG	60.0	86	In house
Myog(NM_031189)	F:GCAATGCACTGGAGTTCG<br>R:ACGATGGACGTAAGGGAGTG	60.0	94	In house
Myf5(NM_008656)	F:ACAGCAGCTTGACAGCATC<br>R:AAGCAATCCAAGCTGGACAC	60.0	85	Ishibashi et al. JCB 171:471 (2005)
Myf6 (NM_008657)	F:GCCTCGTGATAACTGCTAAGG<br>R:GTTCCAAATGCTGGCTGAGT	60.0	162	Rubin et al. Cancer Cell 19:177 (2011)
Gapdh (NM_001289726)	F:GGTGCTGAGTATGTCGTGGA<br>R:ACAGTCTTCTGGGTGGCAGT	60.0	290	In house
Actb (NM_007393.3)	F:GGCTGTATTCCCCTCCATCG<br>R:CCAGTTGGTAACAATGCCATGT	60.0	154	PrimerBank[d]: 6671509a1
**Proviral Integration PCR Primers:**				
SFFV	F: CTGCTTCTCGCTTCTGTT C	64.5	In house	
Kras	R: TGCTAACTCCTGAGCCTGTTTCGT	64.5	In house	
**p53 Genotyping PCR Primers**				
p53 × 6.5	F: ACAGCGTGGTGGTACCTTAT	60.0	Jackson Laboratory[e]	
p53 × 7	R: TATACTCAGAGCCGGCCT	60.0	Jackson Laboratory[e]	
p53xNeo	F: CTATCAGGACATAGCGTTGG	60.0	Jackson Laboratory[e]	

[a:]Abbreviations: ICC: immunocytochemistry, IHC: immunohistochemistry, IB: immunoblot
[b:]Dilution dependent on concentration of protein-specific primary antibodies
[c:]Primer annealing temperature
[d:]http://pga.mgh.harvard.edu/primerbank/
[e:]jaxmice.jax.org/protocolsdb/f?p=116:2:3868241449886939::NO:2:P2_MASTER_PROTOCOL_ID,P2_JRS_CODE:5741,002101

Supplementary Table S3. Log2-transformed normalized gene expression data used for GSEA

Supplementary Table S4. Ranked list of gene expression analysis comparing Kras^{G12D} expressing myoblasts (Kras-H, Kras-L) to empty vector control myoblasts (Control-H, Control-L)

Supplementary Table S5. Ranked list of gene expression analysis comparing all tumors (Kras-H, Kras-L) to empty vector control myoblasts (Control-H, Control-L)

Supplementary Table S6. Ranked list of gene expression analysis comparing all Kras-H tumors to Kras-L tumors

Supplementary Table S7. Ranked list of gene expression analysis comparing Kras-H tumors to Control-H myoblasts

Supplementary Table S8. Ranked list of gene expression analysis comparing Kras-L tumors to Control-L myoblasts
